# Supplementary material for: Acute Myocardial Infarction among Young Adult Men in a Region with Warm Climate: Clinical Characteristics and Seasonal Distribution
Source: Int J Environ Res Public Health. 2020 Aug 24;17(17):6140. doi: 10.3390/ijerph17176140 (PMC7503405; doi:10.3390/ijerph17176140)
Supplement: Supplementary file 1 [file ijerph-17-06140-s001.pdf]

Table S1. Comparison of clinical characteristics between the included cases and the excluded cases

	**The included cases (N=1413)**	**The excluded cases (N=875)**	***P* value**
Age (SD)	64.0(13.7)	68.0(13.1)	<0.001★
The <45y/o (%)	138 (9.8) (n=1413)	54 (6.2) (n=875)	0.003★
CAG (%)	1221 (86.4) (n=1413)	516 (59.0) (n=875)	<0.001★
Number of stenosed vessels			0.952
≤1 (%)	566 (46.4)	240 (46.5)	
≥2 (%)	655 (53.6)	276 (53.5)	
Conventional risk factors			
Smoking (%)	675 (53.1) (n=1413)	249 (49.5) (n=796)	0.106
DM (%)	415 (35.2) (n=1413)	152 (33.1) (n=743)	0.338
Hypertension (%)	757 (37.7) (n=1413)	275 (61.5) (n=749)	0.450
T C (mg/dL)	175.6(46.4) (n=1413)	173.5(50.3) (n=445)	0.414
HDL-C (mg/dL)	40.3(12.6) (n=1413)	40.5(13.6) (n=319)	0.459
TG (mg/dL)	143.0(123.3) (n=1413)	147.9(115.9) (n=438)	0.838
LDL-C (mg/dL)	108.3(39.4) (n=1413)	109.2(66.3) (n=206)	0.795
BMI (kg/m^2)	25.2(3.9) (n=1413)	24.3(4.2) (n=516)	<0.001★

AMI: acute myocardial infarction; CAG: coronary angiography; DM: diabetes mellitus; TC: total cholesterol; TG: triglycerides; HDL-C: high-density lipoprotein cholesterol; LDL-C: low-density lipoprotein cholesterol; BMI: body mass index. ★ indicates P<0.05.
